# Knowledge Generation with Rule Induction in Cancer Omics

**DOI:** 10.3390/ijms21010018

**Published:** 2019-12-18

**Authors:** Giovanni Scala, Antonio Federico, Vittorio Fortino, Dario Greco, Barbara Majello

**Affiliations:** 1 Department of Biology, University of Naples Federico II, 80126 Naples, Italy; barbara.majello@unina.it; 2 Faculty of Medicine and Health Technology, Tampere University, 33014 Tampere, Finland; antonio.federico@tuni.fi (A.F.); dario.greco@tuni.fi (D.G.); 3 Institute of Biomedicine, University of Eastern Finland, 70210 Kuopio, Finland; vittorio.fortino@uef.fi; 4 Institute of Biotechnology, University of Helsinki, 00014 Helsinki, Finland

**Keywords:** rule induction, cancer, omics data, machine learning, TCGA (The Cancer Genome Atlas), patients classification

## Abstract

The explosion of omics data availability in cancer research has boosted the knowledge of the molecular basis of cancer, although the strategies for its definitive resolution are still not well established. The complexity of cancer biology, given by the high heterogeneity of cancer cells, leads to the development of pharmacoresistance for many patients, hampering the efficacy of therapeutic approaches. Machine learning techniques have been implemented to extract knowledge from cancer omics data in order to address fundamental issues in cancer research, as well as the classification of clinically relevant sub-groups of patients and for the identification of biomarkers for disease risk and prognosis. Rule induction algorithms are a group of pattern discovery approaches that represents discovered relationships in the form of human readable associative rules. The application of such techniques to the modern plethora of collected cancer omics data can effectively boost our understanding of cancer-related mechanisms. In fact, the capability of these methods to extract a huge amount of human readable knowledge will eventually help to uncover unknown relationships between molecular attributes and the malignant phenotype. In this review, we describe applications and strategies for the usage of rule induction approaches in cancer omics data analysis. In particular, we explore the canonical applications and the future challenges and opportunities posed by multi-omics integration problems.

## 1. Introduction

During the last century [1], cancer biology has been arguably one of the most investigated research fields. The effort and resources employed to understand the mechanisms of cancer development and progression generated a substantial knowledge of the deregulated physiology of cancer cells and their altered relationships with the surrounding environment. However, such knowledge is still far from sustaining a pharmacological resolution of cancer [2]. The completion of various multi-unit projects, such as The Cancer Genome Atlas (TCGA) and the International Cancer Genome Consortium (ICGC), have boosted cancer research on big data highlighting the complexity of cancer system. As a consequence, mathematical modelling of biological data has become a burgeoning area of cancer research [3], allowing a system-level understanding of the principles governing the structure and behavior of a cancer phenotype [4]. In this context, systems biology and machine learning techniques currently represent the leading actors of system complexity breakdown and inference/prediction of the malignancy outcome. Independent component analysis (ICA), principal component analysis (PCA), and non-negative matrix factorization (NMF) are only some of the standard methods applied to the analysis of high-dimensional biological data [5]. The integration of multi-omics and/or multi-cancer layers is another crucial task in the interpretation of cancer data, especially regarding the characterization of drug sensitivity and the prognosis prediction of oncological patients [6]. Machine learning (ML) has been extensively used to address these challenges in cancer research [7,8].

ML focuses on the progressive improved performance of a computer algorithm for a specific task through its ability to “learn” by the data. A number of machine learning-based approaches have been developed in recent years that learn relationships (as functional associations between a set of features and one or more target variables) between the molecular profiles of cancer patients and clinical outcomes [9,10,11,12,13].

In particular, unsupervised methods have been implemented for the integrative analysis of multi-omics data, while supervised methods have been used for the classification of cancer patients into clinically relevant sub-groups and for the identification of biomarkers for disease risk and prognosis [14,15]. Different techniques, such as logistic regression, naive Bayes, LinearSVC, Support Vector Machines (SVM), random forest and neural network, have been extensively used for cancer classification tasks. In particular, Deep Learning techniques, an evolved form of neural networks, have emerged as a powerful approach for addressing computational challenges in cancer medicine, which can both encode and model many kinds of complex data (e.g., different omics data types, electronic health records and images) both in supervised (e.g., biomarker identification) and unsupervised (e.g., molecular subtype detection) settings [16]. However, as datasets become larger and more complex over time, deep learning methods may begin to identify relationships or patterns in data that are more accurate but difficult to interpret. Furthermore, in translational medicine, it is important to consider the interpretability alongside the accuracy of the extracted models. For instance, it is well-known that samples in cancer genomic studies are often non-representative of the general population of interest, and that cases of disproportionality between the number of measured features and the number of observations may introduce nominally irrelevant features of the data (called ‘leakage’ in other fields [17]). In these cases, the complexity of deep networks makes it difficult to determine when their predictions are likely to be based on such irrelevant features. In practical terms, when using ML methods, it is important to consider the extent to which biases may be learned by the model and whether or not a model is sufficiently interpretable to identify certain biases [18]. A class of ML methods which is not commonly applied to omics data and cancer medicine is rule induction. Rule induction is a ML technique used to extract classification rules -usually in the form IF (conditions) THEN (predicted class)- from data. Rule-based strategies can generally enhance the effectiveness and interpretability of classification models. 

In this paper, we explore and describe the principal applications of rule induction techniques to cancer research. We discuss the past achievements along with the possible strategies to build and set-up rule induction systems capable of capture and represent reliable and clinically significant relationships between molecular and clinical features and cancer related classes.

## 2. Challenges of Omics Data Analysis in Cancer Research

The completion of the Human Genome Project [19] boosted the development and technological advancements of large-scale experiments in order to characterize every aspect of the molecular mechanisms of the cell. These include gene expression, epigenetic modifications (including DNA methylation, histone modification and chromatin architecture), protein expression and quantification of metabolites [20]. With the advent of hybridization-based technologies first (such as the DNA microarrays) and the high-throughput sequencing (HTS) technologies later, the biomedical science community became able to globally characterize the molecular status of the cell in a few hours and at a relatively low cost [21]. As a consequence, an unprecedented insight was given to the etiology and progression of a humongous number of human diseases, including cancer. On the other hand, an enormous amount of data has been produced, making sometimes difficult the analysis and the interpretability of the results. This is even more marked in cancer research, where a notable effort has been spent to understand the underlying molecular basis, as well as to identify molecular biomarkers not only responsible for the initiation and progression of the malignant transformation, but also for the impaired sensitivity of the patients to standard therapeutic strategies [22]. Although a plethora of computational methods have been developed so far, the research community is still far from disentangling the complexity of the malignant transformation with current methods of knowledge extraction from omics data. Apart from the intrinsic complexity of the underlying malignant process, the reasons of this issue are purely technical. First of all, omics data are typically high dimensional, being composed by a high number of measurements (from here on called features) and a relatively small number of samples (hereby called observations). Typically, the throughput size of current omics technologies ranges from thousands to hundreds of thousands of features and an order of hundreds of observations, given the elevated number of features currently accessible at a relatively cheap cost with current technologies. Specifically, the numbers can range from tens of thousands when considering gene expression quantification at isoform level, to hundreds of thousands, considering the most recent epigenomics datasets. On the other hand, until recently, the single studies were limited to tens of sequenced samples in the best cases. To date, the biggest studies have been performed by multi-institutional consortia as well as The Cancer Genome Atlas (TCGA) and the International Cancer Genome Consortium (ICGC). These studies comprise multi-omics experiments for tens to thousands of patients [23]. Although such studies are representative of the oncological patients’ population, the relative dimensionality between the number of features and observations is still considerably unbalanced.

**Figure 1:**
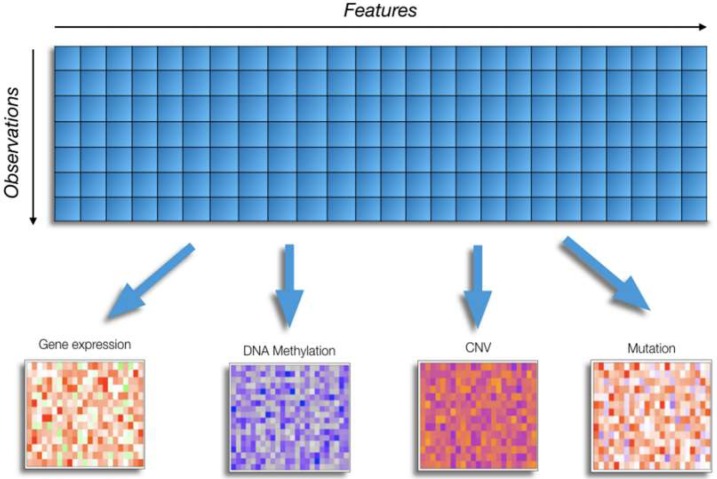
Typical shape of an omics data matrix. Blue arrows link the column of the matrix to the different omics data type that are frequently found in a multi-omics experiment.

This is an intrinsic feature of omics data and confers to omics datasets a typical unbalanced shape toward the features dimension (Figure 1). The main consequence of such unbalanced shape of omics data sets is the so called “curse of dimensionality”. This latter is in turn related with the emergence of spurious associations between features and variables of interest where the increased dimensionality in the number of features and the relatively small sample size, make most ML methods vulnerable to overfitting, i.e., high accuracy on the training data but poor generalization on unseen test data. Altman and Krzywinski [24] describe the effects of the curse of dimensionality in big data on many aspects of the data analysis. In particular, they discuss curse of dimensionality as it applies to data sparsity, multicollinearity, multiple testing and overfitting. Many dimension-reduction methods have been developed so far to deal with such problems such as variable selection and principal component analysis, which can help to reduce dimensionality [24].

Omics data assays are typically constituted of many measurements of numerical (continuous) features. Ideally, one would like to build an omics dataset that is complete (no missing observations), error free (without measurement errors/biases) and consistent. Unfortunately, such assays are subject to measurement as well as to technical biases, especially when multiple experiments performed in different experimental settings.

This latter point particularly emerges when omics datasets are built as set of features collected from multiple studies. In this case, the values of the measurements of the same attribute can differ in representation, scale, and value [25]. 

All of the above cited aspects of omics datasets make knowledge extraction a non-trivial task and many techniques have been developed to face each of the above cited problems. Despite the large amount of techniques devised to address this issue, the ultimate key factor for all of these methods to be effective is the availability of a sufficient number of samples. This is currently becoming a reality, especially in cancer omics, where the amount of produced data in several big scale projects [26,27,28,29] gives the possibility to generate appreciable sized datasets for the investigation of several molecular disorders using different molecular districts.

## 3. Rule Induction

Rule induction algorithms consist of a group of pattern discovery techniques belonging to the class of supervised machine learning. In the supervised machine learning, the main task is to learn reproducible relationships (models) from annotated data. The relationship to be learnt is between a set of predictor variables (features) and one or more target variables, when these target variables correspond to discrete values (or Classes). Once the relationship is learnt, the obtained model can be used to predict the class given the attribute values of a new observation. The task of learning such relationship is called classification. Classification methods can be grouped into two classes, based on the way they represent the learnt model, as symbolic or subsymbolic approaches [30]. In the symbolic representations, the model is explicitly represented using a symbolic formalism that can be directly interpreted by the human. Classical examples of symbolic representations are decision trees and decision rules. In subsymbolic models, the discovered patterns are represented by formalisms and structures that are not directly interpretable by the human. Examples of these formalisms are artificial neural networks and support vector machine (SVM) or ensemble-based approaches like random forest (RF). The choice of using a symbolic approach rather than a subsymbolic one is mainly guided by the work purpose: when the classification is the main goal of the analysis, the method offering best classification performances is usually chosen. In contrast, when the purpose is to extract knowledge from the data, the methods offering the most convenient and intelligible form of representation for the discovered patterns should be preferred.

Induction rule algorithms can be used in combination with statistical learning methods in order to determine descriptive patterns in the form of rules intended for interpretation of the discovered relationships between variables in the data. Statistical learning methods aim to build mathematical models representing relationships between variables (e.g., between predictors and a target/response variable) that can be used to better understand the phenomena underlying the data and to be able to predict future values. However, these mathematical models are often difficult to be interpreted and managed. An efficient way to make statistical learning outcomes more interpretable is to add a further analytical step with the aim of finding more descriptive patterns in the analyzed data. This is the case for neural networks and their advanced version known as deep learning. These machine learning techniques provide formidable classification performance, but at the expense of the interpretability of the hidden classification rules. Indeed, the difficulty in understanding how neural networks define their classification decisions could represent, for instance, a big problem for physicians who often need to find a meaningful explanation for the decisions taken by the employed ML method. To solve this problem, rule extraction strategies can be used in order to extract symbolic rules from neural networks. During the past few years many strategies have been proposed for this laborious computational task [31,32]. Rule extraction can also be applied to ensemble of decision trees [33,34] or other machine learning techniques such as support vector machine [35]. Another interesting application of statistical learning method combined with rule extraction algorithms is given by adaptive neuro-fuzzy inference systems (ANFIS). In this neural network-based strategy the Takagi-Sugeno fuzzy inference system is used to build a set of fuzzy IF-THEN rules aiming to approximate nonlinear functions [36].

Since the main focus of machine learning approaches applied on cancer omics data has been patients (sub-) classification, the semi-symbolic approaches have been widely used over the symbolic ones. With the current number of available datasets, symbolic approaches applied to cancer omics data analysis offer humongous potential.

A widely used symbolic approach is rule learning. Rule learning tries to represent the relationships between features and classes in the form of (a set of) IF-THEN rules. In particular, starting from a set of training samples S = s_1_,s_2_,..., s_n_, where each sample s_i_ is described by a vector of features x_i_ = x_1,i_,x_2,i_,...,x_p,i_ and a class label g_i_, the rule learning determines a set of rules, where each rule can be described as follows: *IF (x_1_ Op val_1_) AND (x_2_ Op val_2_) AND … THEN class g,* where *Op* is a comparison operator.

Examples of successful usage of rule learning methods in cancer research have been reported both on small scale studies [37,38,39,40,41], as well as on large scale projects like TCGA [42,43,44]. 

## 4. Useful Structure of Rules for Cancer Omics Data

Several rule induction methods exist in literature that are suitable for cancer omics data analysis (Table 1, Additional file 1 in Supplementary Materials). For instance, Cestarelli et al. [42], implemented and successfully tested a rule-based method, named CAMUR, able to extract multiple classification models and create a knowledgebase of rules in order to perform reliable classification on cancer data. CAMUR was tested on TCGA gene expression data of invasive breast cancer (BRCA), head and neck carcinoma (HNSC) and stomach cancer (STAD) and validated on independent datasets from the Therapeutically Applicable Research to Generate Effective Treatments (TARGET) program and the Gene Expression Omnibus (GEO) repository. CAMUR successfully identified different gene sets previously associated with the three TCGA cancer types. In a similar approach, Celli and colleagues implemented a method named BIGBIOCL [44], which can apply supervised classification methods to high-dimension datasets. The authors tested their tool on methylation data from three cancer types from TCGA, including breast, kidney and thyroid carcinoma. This method was able to identify as relevant features some of the already known cancer driver genes, as well as *TP53* and *PIK3CA,* or other high-confidence oncogenic candidates. Furthermore, the k-top scoring pairs (k-TSP) method, developed by Aik Choon Tan et al. [40], based on the concept of “relative expression reversals”, was used to generate a set of decision rules involving only a small number of relationships between couples of genes, greatly improving the interpretability in comparison with other learning methods. In their work, the authors tested k-TSP on a Leukemia dataset taken from Golub et al., 1999 [45]. The k-TSP method was able to identify 18 genes which are able to discriminate Acute Myeloid Leukemia (AML) from Acute Lymphoblastic Leukemia (ALL), and 9 of them (*CD33*, *ZYX*, *TCF3*, *CST3*, *ATP2A3*, *CCND3*, *TOP2B*, *CTSD* and *DF*) were already pointed out by Golum and colleagues to be associated with AML and ALL. The choice of the most appropriate method in this context is guided by the main purpose of “generating knowledge from data”.

Keeping this statement into consideration, it is possible to list a series of desired properties for the generated rule set.

- It is important for the algorithm to be stable over the generated rule sets. In particular, one would expect to obtain the same (or at least highly similar) rule sets when running the algorithm on different versions of the learning substrate. This also guarantees that the system is capable of capturing as many as possible of the representable relationships in the chosen formalism. When generating knowledge in cancer studies, it is desirable to generate the complete set only once and to guarantee that it is not dependent on the employed training set.
- The generated rule set, should contain all and only the most relevant relationships, thus keeping the number of rules as lower as possible. This can be obtained by removing the redundant rules and/or the rules covering only a few specific cases of a class.
- The generated rules should be based on all and only the features that are directly related with the predicted variable. In cancer research, the major interest is the identification of key molecular actors useful for (1) the comprehension of the mechanisms leading to the malignant transformation and (2) the identification of therapeutic targets.
- The ideal rule-based model should exhibit highly accurate levels, thus guaranteeing that the set of rules is powerful enough to capture putative relationships between the features and the classes.
- The rule set contains no redundant rules. That means that each association rule of the set must contain information not deducible from other rules
- The obtained rule set should contain all the rules involving relevant features for the phenomenon. Cancer omics data analysis often shows that few features (e.g., expression status of a few genes) are sufficient to classify the disease with appreciable accuracy. Therefore, only one model (e.g., the one with higher accuracy) is usually chosen. In rule induction for knowledge extraction, the aim is not only limited to the classification performances of the model. In this setting, indeed, it is necessary for a rule base to contain as many as possible of the rules involving relevant features, even if this leads to a redundant classification system. Such an approach enables to capture many putative relationships linking causative factors to the phenomenon under study. This approach has been successfully implemented in CAMUR [42] where the rule induction algorithm has been specifically designed to learn alternative and equivalent solutions instead of a single rule set containing few but highly discriminant rules.

## 5. Feature Selection and Representation

Two crucial factors may affect the effectiveness of rule induction for knowledge generation with cancer omics, namely the selection and representation of features.

When dealing with high dimensional data, like cancer omics datasets, the quality of the starting set of features is determinant in the generation of an efficient rule set. This process is generally referred to as feature selection or feature reduction.

When the number of features, like the expression of all the human genes, highly surpass the number of available observations (i.e., samples), it is important if not mandatory to reduce the size of the starting set of features for a correct application of machine learning algorithms.

As discussed before, one possible strategy is to select one of the smallest sets of features guaranteeing the highest classification performances. Alternatively, the maximal set including all the relevant features can be chosen. 

For the purpose of knowledge discovery, the second strategy naturally fits, since the task is to collect as much knowledge in different relationships. Different approaches can be followed to accomplish this task, such as:

- Technical feature selection strategies,
- knowledge-driven feature selection strategies, and
- hybrid feature selection strategies.

Technical feature selection strategies aim to reduce data dimensionality by removing redundant and/or irrelevant features or by transforming the original high-dimensional feature space to an analytically equivalent but smaller set of features. Transformation-based approaches, by irreversibly transforming the data points, have the drawback to destroy the original dataset semantic (or nature). On the other hand, feature elimination seeks to retain the original structure of the data by selecting features. This aspect is particularly useful when feature selection precedes other procedures that require the features to be in their original space, as in rule induction, where the rules need to be human-readable. Feature elimination can be conducted with filter, wrapper or embedded methods. Examples of filter methods include RELIEF [56], as well as correlation and mutual information-based strategies [57]. Wrapper methods, instead, combine search strategies (exhaustive, heuristic or random search) with machine learning methods. Examples of feature selection methods applied to omics data are Boruta [58], VarSelRF (Variable Selection using Random Forests) [59], SVM-RFE (Support Vector Machines – Recursive Feature Elimination) [60], and FPRF (fuzzy pattern – random forest) procedure [61]. In embedded techniques, the feature selection algorithm is integrated as part of the learning algorithm. Example of embedded algorithms are decision tree algorithms (ID3 [62], C4.5 [46], and CART (Classification And Regression Tree) [63]), and LASSO (Least Absolute Shrinkage and Selection Operator) [64], Ridge (Tikhonov regularization) [65], and ElasticNet [66] when constructing linear models.

The selection of relevant features in knowledge-driven feature selection strategies is based on prior knowledge related to the classification problem. Unlike many other fields, cancer has been widely studied for a long time, and therefore, a humongous amount of prior knowledge exists about potentially relevant biological pathways and related genes (or features). The most common knowledge-based strategy aims at incorporating pathway information into classic feature selection techniques in order to identify features that likely play key roles in cancer-relevant pathways. Examples of this technique are the gene-set analysis-based reduction algorithm (SAMGSR) [67], the reweighted recursive feature elimination (RRFE) method [68] and the generalized elastic net (GELnet) method [69]. 

Another strategy for knowledge driven feature selection is based on the incorporation of all the features that have been reported to be associated with certain relevance in literature with a particular type of cancer or with cancer in general. 

While the technical feature selection approach has the advantage to be not influenced or limited by prior knowledge and thus the possibility to select relevant features not (yet) characterized in the field, the knowledge driven approach has the advantage to be not limited by the information content of the starting sample set and by the analytical power (e.g., ability to capture nonlinear relationships) of the selection models, thus presenting the possibility to include known important factors in the training set.

Hybrid feature selection strategies [70,71] include both technical and knowledge driven approaches. A possible implementation of this strategy is to complete the set of technically discovered features with missing (possibly non redundant) knowledge-based features. For example, when using transcriptomics-based features for cancer classification, one could integrate genes from literature not reported in the technical selection output, and then refine if necessary, the set for redundant (e.g., linearly correlated) features. This approach clearly increases the power of rule induction methods in this setting, but also needs to be performed paying particular attention at keeping the features set within acceptable sizes. 

An additional important aspect in rule discovery applied to omics data is the representation space of the features. As stated before, omics data analyses usually deal with numerical (often continuous) features. Moreover, these values do suffer from measurement errors, batch effects, different scales [25,72,73,74,75].

The goal of discretization is to reduce the number of different values that a continuous feature can assume by grouping them into a relatively small number of intervals or bins. 

The usage of discretized features allows to work with the majority of rule extraction methods that assume discrete values and generally requires less resources in terms of space and computational time. 

On the other hand, the discretization of continuous variables can cause, in some cases, loss of information compared to the available original data [76].

Different discretization methods exist in literature and they mainly differ in the way the discrete classes are defined and in the employed mapping function used to map values from the numerical space to the discretized space. For a complete review of discretization methods applied to omics data, see [77].

It is well-known that omics data contain noise due to experimental procedures and biological heterogeneity. An efficient way to reduce the negative effect of noise is to apply fuzzy discretization, using a fuzzy inference system [78]. This technique has been applied to identify set of discriminant genes from gene expression data [79]. The main idea is to transform continuous variables into fuzzy labels, by estimating membership functions for each gene from the gene expression values across all the samples. Each membership function will represent the range of variability of a given fuzzy label (e.g., high, medium or low expression). Subsequently, the fuzzy membership functions are used to discretize the gene expression values in each sample.

In the first step, given a set of n expressed sequence tags (ESTs) or genes belonging to m microarrays, the discretization process is based on determining the membership function of each gene to the previously linguistic labels. 

Standard discretization methods assign classes in a strict way, meaning that one and only one class is typically assigned to each different value of each attribute. It thus happens that very close numeric values end up in different classes or that uncertain values are forced to belong to a class they do not belong to.

To overcome this problem, fuzzy classes can be used instead of discrete classes, in a mapping process called fuzzification [50]. 

Mapping from a numeric variable to a fuzzy one, constituted of *n* fuzzy classes, essentially consists in assigning a vector of probabilities to each observation, representing the degree of belief for the observation to belong to each class. This approach has been successfully applied in cancer omics-based classification in [50] to classify different cancer forms using gene expression data. 

## 6. Rule Base Evaluation

Another important aspect of rule-based models for cancer omics is the choice of a suitable set of evaluation metrics to quantify the amount of information that the system has been able to learn from the data.

Such metrics can be used both to provide an estimate of the quality of the models as well as a method of choice between alternative models available for the same task.

In the case of rule induction for knowledge generation in cancer, the following two metrics can be used:

- Coverage of cases (classification oriented): namely the fraction of observations from the training set whose features’ values satisfies at least one rule. This metric summarizes the fractions of different learnt relationships driving each class from the input features. The extremely high value of this indicator should also be treated with caution since they can be an index of overfitting.
- Coverage of features (knowledge discovery oriented): namely the fraction of relevant features that appear in at least one rule. This metric is particularly important in cancer rule induction, since the main interest in this activity is to explore as many relationships as possible that link the relevant molecular features with the phenotype of interest.

## 7. Knowledge Representation

As stated before, the output of a rule induction algorithm is a set of rules (the rule base) of the form:

IF (x_1_ Op_i_ val_1_) AND (x_2_ Op_j_ val_2_) AND … THEN class g_i_, where x_i_ ∈ x_1_, x_2_,..., x_p_ are the considered features, g_i_ is a class label, and Op_x_ is a comparison operator. 

The shape of a rule set can usually be improved by applying a series of post-processing steps before making it available to the user. The post-processing operation does not alter the rule base semantics in terms of features/class relationships, but instead operates on rules in order to simplify their structure and/or reduces the size of the rule set by eliminating redundant rules (pruning) or grouping rules based on common features (grouping).

After a rule base has been produced, assessed, and post-processed, a convenient representation strategy is needed in order to consult the obtained knowledge. A possible representation format is given by decision trees, and their generalization to graph structures, while other approaches include decision tables and flowcharts.

The exploration of single rules or groups of rules can become a complex task, especially when their number is relatively large. In this latter case, specific interfaces, mediating the interaction between the user and the rule base need to be built. In two recent works, by applying rule induction to TCGA gene expression data [42,43], 21 rule sets, for as many different cancer datasets, were generated. In order to facilitate knowledge extraction from this sets, the authors proposed five different kinds of query: i) A gene list, to query the list of features used in the rule set of a given cancer type, ii) literals and conjunctions list, to retrieve the most relevant literals (e.g., geneA > 20) and conjunctions (e.g., geneA > 0.4 & geneB < 50) for a given cancer type, along with the number of instances classified by rules containing them, iii) rules list, to recover the list of all the rules derived for a given cancer type along with the corresponding measures of reliability, iv) literals statistic, to retrieve literals (i.e., genes) with occurrence in a given cancer within a specific frequency range, and v) gene pairs, to retrieve the gene pairs that occur more frequently in a rule set of a given cancer type. 

All of the above rules are just an example of the multiple ways that can be provided to the investigators to extract information from a rule set. More complex queries can be built both to explore as well as to navigate a rule set. Mixing the graphical representation of rule sets and user queries is one way to facilitate and boost this operation.

## 8. Integrating Multi-Omics and Non-Omics Features

For many cancer datasets, the current literature provides concurrent assays of different molecular districts that could be integrated as molecular features in rule induction procedures. The usage of omics data from different molecular layers does not only improve the classification performances of the obtained models but also provides an improvement in terms of acquired knowledge [80]. In a knowledge-oriented setting, the application of such methods to cancer data would help to discover cross-omics molecular relationships that are pivotal in the understanding of the diseases’ mode of action. One interesting approach of the application of rule induction to a classification task based on multiple molecular layers is given in [81], wherein the authors combined genome wide methylation data and genome wide assays of 20 different histone modification signals to classify the expression status of human genes in CD4+ T cells. In particular, their system was able to derive a rule base of 83 different multi-omics rules reflecting the possible interactions between methylation and histone modifications in the modulation of gene expression. Another interesting example of the application rule induction to cancer classification using features from different omics layers is given in [82]. Here, the authors generated rule sets discriminating between cancer and normal samples from three different TCGA tumors (breast invasive carcinoma, thyroid carcinoma, kidney renal papillary cell carcinoma using features derived from mRNA expression data and DNA methylation. In particular, by applying four different rule induction algorithms (C4.5, Random Forest, RIPPER, and CAMUR), the authors showed that the models obtained using an integrated feature set contain several genes and relationships that the systems were not able to capture when using single omics.

Moreover, extending the integration from different omics data to clinical features could represent a further improvement to rule induction models for cancer data. In fact, this would give the possibility to produce unique and very useful rule bases, able to explain the relationships between clinical properties and molecular entities from different aspects in the determination of the malignant phenotype.

## 9. Conclusions

Herein, we addressed the application of rule induction algorithms to molecular cancer research for generating knowledge explaining the complex relationships that link omics and clinical data in human cancer. 

The vast majority of machine learning applications that use this kind of data are mainly devoted to classification tasks, having the classifier itself as their main outcome. We described the opportunity and the potential of applying methodologies focused on the generation of an intelligible decisional process, rather than black box classifiers. 

We addressed the general problems to face up when applying these methods to cancer omics along with the main steps and existing techniques to properly address each one of them.

Finally, we discussed the possibility and the potential benefits deriving from the application of rule induction on features from multiple molecular as well as clinical layers to build rule sets capable of showing relationships involving clinical and molecular factors at the same time.

In cancer research, the discovery of multi-omics relationships is an essential step in the study of many aspects, ranging from disease exploration to the discovery of novel therapeutic targets. 

Rule induction algorithms, like many other pattern discovery systems, have their limitations in both the number as well as the complexity of the relationships that they are able to model. Even with these limitations, the combination of their explanatory power with the current availability of data, can bring to the field an unprecedented amount of novel information to explore, integrate, and exploit in the study of cancer diseases.

## Figures and Tables

**Table 1:** Rule induction methods applicable to cancer omics data.

Tool	Strategy	Output	Implementation	Language
C4.5 [46]	Decision tree	Decision trees	WEKA [47]/J48	Java, R, Python
RIPPER (Repeated Incremental Pruning to Produce Error Reduction) [48]	Sequential covering	Rule set	WEKA/JRip	Java, R, Python
PART (Partial Decision Trees) [49]	Sequential covering	Rule set	WEKA/PART	Java, R, Python
CAMUR (Classifier with Alternative and MUltiple Rule-based models) [42,43]	Sequential covering	Rule set	CAMUR website ^1,2^	Java
BIGBIOCL [44]	Sequential covering	Rule set	BIGBIOCL github ^3^	Java
FURIA (Fuzzy Unordered Rule Induction Algorithm) [50]	Sequential covering	Fuzzy rule set	WEKA/FURIA	Java, R, Python
MLRules (Maximum Likelihood Rule Ensembles) [51]	Sequential covering and probability estimation	Rule set	MLRules website ^4^	Java
LERS (Learning from Examples based on Rough Sets) [52]	Rough set theory	Rule set	R/RoughSets package	R
TSP (Top Scoring Pairs) [39]	Rank based	Rule set	R/tspair package	R
k-TSP (k - Top Scoring Pairs) [40]	Rank based	Rule set	R/switchbox package	R
BIOHEL (Bioinformatics-oriented Hierarchical Evolutionary Learning) [53]	Evolutionary rule learning	Rule set	BIOHEL website ^5^	C++
CN2-SD (Clark & Niblet – Subgroup Discovery) [54]	Subgroup discovery	Rule set	CN2-SD website ^6^	Java
SDEFSR (Subgroup Discovery with Evolutionary Fuzzy Systems) [55]	Subgroup discovery	Fuzzy rule set	R/SDEFSR	R

^1^http://dmb.iasi.cnr.it/camur.php; ^2^http://bioinformatics.iasi.cnr.it/camurweb/home; ^3^https://github.com/fcproj/BIGBIOCL; ^4^http://www.cs.put.poznan.pl/wkotlowski/software-mlrules.html; ^5^https://ico2s.org/software/biohel.html; ^6^http://www.keel.es/algorithms.php.

## Supplementary Materials

Supplementary materials can be found at https://www.mdpi.com/1422-0067/21/1/18/s1.

## Abbreviations

TCGA	The Cancer Genome Atlas
ICGC	International Cancer Genome Consortium
ML	Machine learning
SAMGSR	Gene-set analysis-based reduction algorithm
RRFE	Reweighted Recursive Feature Elimination
GELnet	Generalized Elastic Net
ESTs	Expressed Sequence Tags
ICA	Independent Component Analysis
PCA	Principal Component Analysis
NMF	Non-negative Matrix Factorization
HTS	High-Throughput Sequencing
SVM	Support Vector Machine
RF	Random Forest
ANFIS	Adaptive Neuro-Fuzzy Inference Systems
RIPPER	Repeated Incremental Pruning to Produce Error Reduction
PART	Partial Decision Trees
CAMUR	Classifier with Alternative and MUltiple Rule-based models
FURIA	Fuzzy Unordered Rule Induction Algorithm
MLRules	Maximum Likelihood Rule Ensembles
LERS	Learning from Examples based on Rough Sets
TSP	Top Scoring Pairs
k-TSP	k- Top Scoring Pairs
BIOHEL	Bioinformatics-oriented Hierarchical Evolutionary Learning
CN2-SD	Clark & Niblet–Subgroup Discovery
SDEFSR	Subgroup Discovery with Evolutionary Fuzzy Systems
VarSelRF	Variable Selection using Random Forests
SVM-RFE	Support Vector Machines–Recursive Feature Elimination
FPRF	fuzzy pattern–random forest
CART	Classification And Regression Tree
LASSO	Least Absolute Shrinkage and Selection Operator
Ridge	Tikhonov regularization
