# Supplementary material for: Knowledge Generation with Rule Induction in Cancer Omics
Source: Int J Mol Sci. 2019 Dec 18;21(1):18. doi: 10.3390/ijms21010018 (PMC6981587; doi:10.3390/ijms21010018)
Supplement: Supplementary file 1 [file ijms-21-00018-s001.pdf]

List of rule induction tools presented in the manuscript

The tools reported in this document are grouped by algorithmic approach. For each tool, a brief description of the strategy is reported along with corresponding references and available implementations. This list of tools for rule induction, is not meant to be exhaustive but can be used as a starting point for users approaching the problem. Almost all of the following tools have been applied in literature on cancer omics datasets.

Decision tree based

C4.5

C4.5 [1] builds a decision tree from a training set by using an extension of Quinlan's ID3 algorithm. The decision tree consists of branches, indicating the results of tests conducted on selected attributes, and leaf nodes, which are assigned to class labels. After building the decision tree, C4.5 tries to reduce its complexity by applying a pruning step, aiming to remove branches with minimum contribution to the overall accuracy. Once the tree has been pruned, knowledge can be extracted and presented in the form of (if-then) rules.
One of the most used implementations of C4.5 is the J48 algorithm implemented in the WEKA [2] suite.
Implementation (language): Weka (Java); Rweka (R); python-weka-wrapper (Python).

Grow & prune algorithms

RIPPER

RIPPER (Repeated Incremental Pruning to Produce Error Reduction) [3] is one of the most efficient and used rule learning algorithms. It implements a divide-and-conquer strategy to rule induction. Ripper applies the so called Incremental Reduced Error Pruning (IREP) to compile an initial set of rules for each class. Then, an additional optimization step considers each rule in the current set in turn and creates two alternative rules from them: a replacement rule and a revision rule. After that, a decision is made on whether the model should keep the original rule, the replacement, or the revision rule, based on the minimum description length criterion.
A widely used implementation of RIPPER is the JRip algorithm from the WEKA suite.
Implementation (language): Weka (Java); Rweka (R); python-weka-wrapper (Python).

PART

PART (Projective Adaptive Resonance Theory) [4] is a partial decision tree algorithm. In particular, PART generates a set of rules according to the divide-and-conquer strategy, removes all instances from the training collection that are covered by this rule and proceeds recursively until no instance remains. To generate a single rule, PART builds a partial C4.5 decision tree for the current set of instances and selects the leaf with the largest coverage as the new rule. Afterwards, the partial decision tree along with the instances covered by the new rule are removed from the training data, in order to avoid early generalization. The process is repeated until all instances are covered by extracted rules.
PART is implemented as well in the WEKA suite.

Implementation (language): Weka (Java); Rweka (R); python-weka-wrapper (Python).

CAMUR

CAMUR (Classifier with Alternative and MUltiple Rule-based models) [5,6] is based on the RIPPER algorithm. It extracts multiple and equivalent rule bases by iteratively computing a rule-based classification model. CAMUR includes an ad-hoc knowledge repository (database) and querying tool.

CAMUR is implemented as a standalone java application and a web application available at http://dmb.iasi.cnr.it/camur.php.

Implementation (language): (Java/Web).

BIGBIOCL

BIGBIOCL (CAMUR improved implementation) [7] is an improved version of CAMUR designed to handle hundreds of thousands of features. According to CAMUR strategy, it is designed to learn multiple alternative and equivalent classification models through iterative deletion of selected features. BIGBIOCL is implemented as a standalone Java application.

Implementation (language): (Java).

Based on fuzzification:

FURIA

FURIA (Fuzzy Unordered Rule Induction Algorithm) [8] is an improved version of the RIPPER algorithm. FURIA uses a modified RIPPER algorithm as a basis and learns fuzzy rules and unordered rule set. The main strength of this algorithm is the rule stretching method, that solves the pressing problem of new records that when classified could be outside the space covered by the previously induced rules. The representation of fuzzy rules is also advanced, essentially, a fuzzy rule is obtained through replacing intervals by fuzzy intervals, namely fuzzy sets with trapezoidal membership function.

FURIA is implemented in Java as a module of the WEKA suite.

Implementation (language): Weka (Java).

Based on probability estimation

MLRules

MLRules (Maximum Likelihood Rule Ensembles) [9] is an induction algorithm for solving classification problems via probability estimation. The basic idea is to exploit the single rules as individual classifiers and then implement upon them an ensemble classification system. Differently from classic sequential covering procedures (also known as divide-and-conquer approaches), new rules are added without adjusting those that have already been added. The main advantage of the MLRules algorithm is given by the fact that a simple and powerful statistical technique is used to induce the rules, which in turn lead to final ensembles with very high prediction accuracy. MLRules is implemented in Java as a module of the WEKA suite.

Implementation (language): Weka (Java).

Rough set theory

LERS (LEM1, LEM2, MLEM2)

LERS (Learning from Examples using Rough Sets) [10] uses the rough set theory to handle inconsistencies in decision rules. The rough set theory is used to obtain the approximation of lower and upper spaces of a crispy set representing a concept. Then, these approximations are used to build two different sets of rules: certain and possible. LERS applies a bottom-up strategy in order to define rules incrementally. At each iteration, it identifies the certain rules and combine the remain possible rules to get the next set of certain and possible rules. This process ends when there are no possible rules to be built. LERS represents a general approach to in knowledge acquisition problem. However, its use in machine learning needs and additional module or computational step aiming to learn a discriminant description [13], i.e., to learn the smallest set of minimal rules, describing the concept. Currently, there are three algorithms for this step: LEM1, LEM2 and MLEM2.

Rank based

TSP

TSP (Top Scoring Pair) [11] is a rule induction technique based on relative values between pairs of features. TSP has been developed for microarray data and build rules on a feature space constituted by pairwise comparisons of gene expression levels. The main advantage of the TSP approach is that, being based on relative values leverages the problem of integrating data from different source that is potentially represented in different scales and can suffer from batch effects. In addition, the TSP classifier provides decision rules that are easy to interpret since they involve relative values between pairs of features (genes in its case). TSP is implemented in R language and available from the tspair Bioconductor package.

Implementation (language): R/tspair package (R).

k-TSP

K-TSP [12] is an extension of the TSP algorithm, which uses exactly k pairs of genes for classifying gene expression data. Instead of using a single comparison a literal, K-TSP uses groups of k comparisons and applies a majority voting among them to decide the truthiness of the complex literal. When k = 1, the algorithm is equivalent to the TSP algorithm. k-TSP is implemented in R language and available from the switchbox Bioconductor package.
Implementation (language): R/switchbox package (R).

Genetic Algorithm based

BIOHEL

BioHEL (Bioinformatics-Oriented Hierarchical Learning) [13] is an evolutionary machine learning system designed to handle with large-scale bioinformatic datasets. BioHEL employs the Iterative Rule Learning (IRL) paradigm. The IRL procedure begins with an empty rule set and the complete set of observations as input and evolves rules one at the time using a genetic algorithm. Each time a rule is evolved by system it is added to the current rule set and all observations covered by the rule are removed from the training set. By iterating this process, rules are added to the set of rules until

all the samples in the training set are covered. BioHel is implemented as a standalone tool in C++ language and can be run by serial execution in CPU mode or by the parallel execution on GPUs. Implementation (language): http://icos.cs.nott.ac.uk/software/biohel. html (C++).

Subgroup Discovery

CN2-SD

CN2-SD algorithm [14] finds rules covering subsets of the population that are sufficiently large and "statistically unusual".

It works iteratively, searching in each iteration for a set of relationships between features (a complex) that covers a large number of examples of a single class and/or other classes. Having found a good complex, the algorithm removes covered observations form the training set and adds the corresponding rule(s) to the rule set. The procedure is repeated iterates until no more satisfactory complexes can be found. CN2-SD is implemented in Java as a module of the KEEL suite.
Implementation (language): KEEL (Java).

SDEFSR

SDEFSR (Subgroup Discovery with Evolutionary Fuzzy System) [15] is a collection of rule induction algorithms based on subgroup discovery that make use of fuzzy logic improve the interpretability of results. SDEFSR algorithms are able to evolve fuzzy rules and use fuzzy set definitions. SDEFSR algorithms are implemented in R language and available from the SDEFSR CRAN package.
Implementation (language): R/ SDEFSR package (R).

References

1. Quinlan, J.R. C4.5: Programs for Machine Learning. Morgan Kaufmann Publishers: Burlington, MA, USA. **1993**.
2. Frank, E.; Hall, M.A.; Witten, I.H. The WEKA Workbench. *Online Appendix for "Data Mining: Practical Machine Learning Tools and Techniques"*, Fourth Edition, Morgan Kaufmann Publishers: Burlington, MA, USA, **2016**.
3. Cohen, W.W. Fast effective rule induction. In the Proceeding of Proceeding of the Twelfth International Conference of Machine Learning, Tahoe City, CA, USA, July 9–12, 1995; Morgan Kaufmann Publishers: Burlington, MA, USA, **1995**, pp: 115–123.
4. Eibe F.; Witten I.H. Generating Accurate Rule Sets Without Global Optimization. Fifteenth International Conference on Machine Learning **1998**, 144–151.
5. Cestarelli, V.; Fiscon, G.; Felici, G.; Bertolazzi, P.; Weitschek, E. CAMUR: Knowledge extraction from RNA-seq cancer data through equivalent classification rules. *Bioinformatics* **2016**, *32*, 697–704.
6. Weitschek, E.; Lauro, S.D.; Cappelli, E.; Bertolazzi, P.; Felici, G. CamurWeb: A classification software and a large knowledge base for gene expression data of cancer. *Bmc Bioinform.* **2018**, *19*, 354.
7. Celli, F.; Cumbo, F.; Weitschek, E. Classification of Large DNA Methylation Datasets for Identifying Cancer Drivers. *Big Data Res.* **2018**, *13*, 21–28.
8. Gasparovica, M.; Aleksejeva, L. Using Fuzzy Unordered Rule Induction Algorithm for cancer data classification. *Mendel* **2011**, 141–147.
9. Dembczyński, K.; Kotłowski, W.; Słowiński, R. Maximum likelihood rule ensembles. *Proceedings of the 25th International Conference on Machine Learning (ICML 2008)* **2008**, Helsinki, Finland.
10. Grzymala-Busse, J.W. A local version of the MLEM2 algorithm for rule induction. *Fundam. Inform.* **2010**, *100*, 1–18.
11. Geman, D.; d'Avignon, C.; Naiman, D.; Winslow, R. Classifying Gene Expression Profiles from Pairwise mRNA Comparisons. *Stat. Appl. Genet. Mol. Biol.* **2004**, *3*, 1–19.

12. Tan, A.C.; Naiman, D.Q.; Xu, L.; Winslow, R.L.; Geman, D. Simple decision rules for classifying human cancers from gene expression profiles. *Bioinformatics* **2005**, *21*, 3896–3904.
13. Bacardit, j.; Burke, E.K.; Krasnogor, N. Improving the scalability of rule-based evolutionary learning. *Memetic Computing*, **2009**, *1*, 55–67.
14. Lavrač, N.; Kavšek, B.; Flach, P.; Todorovsky, L. Subgroup Discovery with CN2-SD. *J. Mach. Learn. Res.* **2004**, *5*, 153–188.
15. García, Á.; Charte, F.; González, P.; Carmona, C.; Jesus, M. Subgroup Discovery with Evolutionary Fuzzy Systems in R: The SDEFSR Package. *R J.* **2016**, *8*, 307.
